# 1α,6β,7β,14β,15β-Penta­hydr­oxy-7α,20-ep­oxy-*ent*-kaur-16-ene

**DOI:** 10.1107/S1600536809052490

**Published:** 2009-12-12

**Authors:** Chuang Feng, Fu-Lin Yan, Xue-Mei Di, Peng-Li Sun

**Affiliations:** a School of Pharmacy, Xinxiang Medical University, Xinxiang, Henan 453003, People’s Republic of China

## Abstract

The title compound, enmenol, C_20_H_30_O_6_, a natural *ent*-kaurane diterpenoid, comprises five fused rings, four of which are six-membered. Cyclo­hexane ring *A* adopts a chair conformation, rings *B* and *C* adopt boat conformations, while ring *D* has an envelope conformation, and two intramolecular O—H⋯O interactions occur. In the crystal, inter­molecular O—H⋯O hydrogen bonds generate a two dimensional network.

## Related literature

For the genus *Isodon* and diterpenoids from this genus see: Sun *et al.* (2001 ▶); Mori *et al.* (1970 ▶); Wang *et al.* (1995 ▶); Yan *et al.* (2008 ▶). For bond-length data, see: Allen *et al.* (1987 ▶).
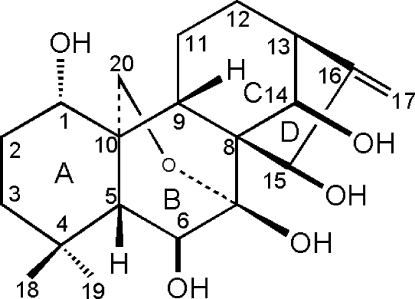


## Experimental

### 

#### Crystal data

C_20_H_30_O_6_
*M* *_r_* = 366.44
Orthorhombic, 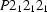

*a* = 8.0007 (3) Å
*b* = 10.7161 (6) Å
*c* = 20.7759 (9) Å
*V* = 1781.25 (14) Å^3^
*Z* = 4
Mo *K*α radiation
μ = 0.10 mm^−1^
*T* = 93 K
0.43 × 0.37 × 0.23 mm

#### Data collection

Rigaku AFC10 Saturn724+ diffractometer
14459 measured reflections
2336 independent reflections
2263 reflections with *I* > 2σ(*I*)
*R*_int_ = 0.029

#### Refinement

*R*[*F* ^2^ > 2σ(*F* ^2^)] = 0.031
*wR*(*F* ^2^) = 0.071
*S* = 1.01
2336 reflections
249 parameters
H atoms treated by a mixture of independent and constrained refinement
Δρ_max_ = 0.28 e Å^−3^
Δρ_min_ = −0.17 e Å^−3^

### 

Data collection: *CrystalClear* (Rigaku, 2008 ▶); cell refinement: *CrystalClear*; data reduction: *CrystalClear*; program(s) used to solve structure: *SHELXS97* (Sheldrick, 2008 ▶); program(s) used to refine structure: *SHELXL97* (Sheldrick, 2008 ▶); molecular graphics: *SHELXTL* (Sheldrick, 2008 ▶); software used to prepare material for publication: *SHELXTL*.

## Supplementary Material

Crystal structure: contains datablocks global, I. DOI: 10.1107/S1600536809052490/zs2022sup1.cif

Structure factors: contains datablocks I. DOI: 10.1107/S1600536809052490/zs2022Isup2.hkl

Additional supplementary materials:  crystallographic information; 3D view; checkCIF report

## Figures and Tables

**Table 1:** Hydrogen-bond geometry (Å, °)

*D*—H⋯*A*	*D*—H	H⋯*A*	*D*⋯*A*	*D*—H⋯*A*
O3—H3*O*⋯O6^i^	0.80 (3)	2.00 (3)	2.7761 (18)	164 (3)
O4—H4*O*⋯O5	0.83 (3)	1.95 (3)	2.6806 (18)	146 (2)
O5—H5*O*⋯O2^ii^	0.86	1.90	2.7455 (18)	167
O6—H6*O*⋯O3	0.83 (2)	1.88 (2)	2.6642 (18)	157 (2)

Symmetry codes: (i) 
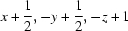
; (ii) 
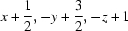
.

**Fig. 1.**
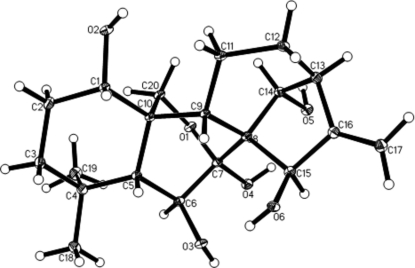
Molecular configuration and atom numbering scheme for compound (I). Displacement ellipsoids are drawn at the 50% probability level.

**Fig. 2.**
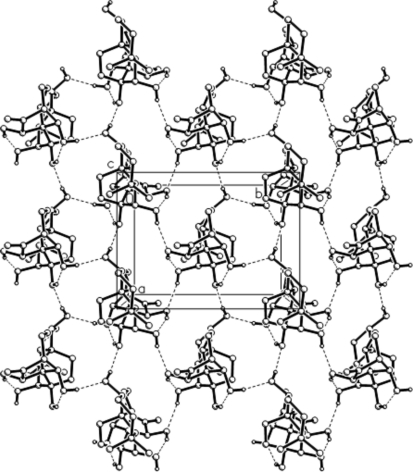
A perspective view of the crystal packing of (I) showing the intermolecular hydrogen bonds as dashed lines.

## supplementary crystallographic information

### Comment

The title compound, 1α,6β,7β,14β,15β-pentahydroxy-7α,20- epoxy-*ent*-kaur-16-ene, C_20_H_30_O_6_ (I) (enmenol) is a natural *ent*-kaurane diterpenoid isolated from the medicinal plant *Isodon japonica* var glaucocalyx (Maxim.) Hara. The leaves of this plant have been used for the treatment of colds, throat swelling and pain, tonsillitis, gastritis, hepatitis, mastitis and cancer. The title compound has also been isolated from *Isodon trichocarpus* (Mori *et al.*, 1970) and *Isodon macrocabyx* (Wang *et al.*,1995), and its structure was postulated from spectroscopic methods (Mori *et al.*, 1970; Wang *et al.*, 1995). The X-ray crystallographic analysis of (I) confirms this proposed molecular structure (Fig. 1). In the structure there is a *trans* junction between ring *A* (C1—C10) and ring *B* (C5—C10). *Cis* junctions are present between ring *B* and ring *C* (C8/C9/C11—C14) and ring *C* and ring *D* (C8/C13—C16). Ring *A* adopts a chair conformation with an average torsion angle of 51.8 (2)°. Rings *B* and *C* adopt a boat conformation because of the formation of the oxygen bridge between C7 and C20. Ring *D* shows an envelope conformation. In addition, the six-membered rings O1/C20/C10/C5—C7 and O1/C7—C10/C20 both adopt boat conformations. The five hydroxy groups at C1,C6,C7,C14 and C15 adopt α,β, β,β,β-orientations respectively. Bond lengths and angles are within expected ranges (Allen *et al.*, 1987), with average values (Å): C*sp*^3^—C*sp*^3^ = 1.544 (2), C*sp*^3^—C*sp*^2^ = 1.514 (2), C*sp*^2^—C*sp*^2^ (CC) = 1.322 (2), C*sp*^3^—O = 1.441 (2).

The title compound has ten chiral centers at C1(*S*), C5(*R*), C6(*S*), C7(*S*), C8(*R*), C9(*S*), C10(*S*), C13(*S*) C14(*R*) and C15(*R*). Although the absolute configuration could not be reliably determined from anomalous dispersion effects, the negative optical rotation showed that this compound belongs to the *ent*-kaurane series as found in genus *Isodon* (Sun *et al.*, 2001), rather than to the kaurane series, allowing us to assign the correct configuration. In the crystal structure, intermolecular O—H···O hydrogen bonds (Table 1) are effective in the stabilization of the structure and are responsible for the formation of a two-dimensional network (Fig. 2).

### Experimental

The dried and crushed leaves of *Isodon japonica* var. glaucocalyx (21 kg), (collected from Huixian Prefecture, Henan Province, China) were extracted three times with Me_2_CO/H_2_O (7:3, *v*/*v*) at room temperature over a period of seven days. The extract was filtered and the solvent was removed under reduced pressure. The residue was then partitioned between water and AcOEt. After removal of the solvent, the AcOEt residue was separated by repeated silica gel (200–300 mesh) column chromatography and recrystallization from Me_2_CO/MeOH (10:1), giving 28 mg of compound (I) (m.p. 527–529 K; optical rotation: [α]_D_^22^ -30° (c 0.15, MeOH). Crystals suitable for X-ray analysis were obtained by slow evaporation of a solution of the compound in MeOH at room temperature.

### Refinement

Hydroxy H atoms were located by difference methods and were included with positional and isotropic displacement parameters refining. With H50 the positional parameters were fixed in the final cycles of refinement. All other H atoms were included in calculated positions and refined as riding atoms, with C—H = 0.98Å (CH_3_), 0.99Å (CH_2_), and 1.00Å (CH), and with *U*_iso_(H) = 1.2 *U*_eq_(C). In the absence of significant anomalous scattering effects, Friedel pairs were merged. The choice of enantiomer was based on comparison of the optical rotation with that of related compounds having known stereochemistry.

### Crystal data

**Table:**

C_20_H_30_O_6_	*D*_x_ = 1.366 Mg m^−^^3^
*M**_r_* = 366.44	Melting point = 527–529 K
Orthorhombic, *P*2_1_2_1_2_1_	Mo *K*α radiation, λ = 0.71073 Å
Hall symbol: P 2ac 2ab	Cell parameters from 6155 reflections
*a* = 8.0007 (3) Å	θ = 3.2–27.5°
*b* = 10.7161 (6) Å	µ = 0.10 mm^−^^1^
*c* = 20.7759 (9) Å	*T* = 93 K
*V* = 1781.25 (14) Å^3^	Block, colorless
*Z* = 4	0.43 × 0.37 × 0.23 mm
*F*(000) = 792	

### Data collection

**Table:**

Rigaku AFC10 Saturn724+ diffractometer	2263 reflections with *I* > 2σ(*I*)
Radiation source: rotating anode	*R*_int_ = 0.029
graphite	θ_max_ = 27.5°, θ_min_ = 3.2°
Detector resolution: 28.5714 pixels mm^-1^	*h* = −10→10
ω scans	*k* = −9→13
14459 measured reflections	*l* = −26→26
2336 independent reflections	

### Refinement

**Table:**

Refinement on *F*^2^	Primary atom site location: structure-invariant direct methods
Least-squares matrix: full	Secondary atom site location: difference Fourier map
*R*[*F*^2^ > 2σ(*F*^2^)] = 0.031	Hydrogen site location: inferred from neighbouring sites
*wR*(*F*^2^) = 0.071	H atoms treated by a mixture of independent and constrained refinement
*S* = 1.01	*w* = 1/[σ^2^(*F*_o_^2^) + (0.0336*P*)^2^ + 0.69*P*] where *P* = (*F*_o_^2^ + 2*F*_c_^2^)/3
2336 reflections	(Δ/σ)_max_ < 0.001
249 parameters	Δρ_max_ = 0.28 e Å^−^^3^
0 restraints	Δρ_min_ = −0.17 e Å^−^^3^

### Special details

**Table:**

Geometry. All e.s.d.'s (except the e.s.d. in the dihedral angle between two l.s. planes) are estimated using the full covariance matrix. The cell e.s.d.'s are taken into account individually in the estimation of e.s.d.'s in distances, angles and torsion angles; correlations between e.s.d.'s in cell parameters are only used when they are defined by crystal symmetry. An approximate (isotropic) treatment of cell e.s.d.'s is used for estimating e.s.d.'s involving l.s. planes.
Refinement. Refinement of *F*^2^ against ALL reflections. The weighted *R*-factor *wR* and goodness of fit *S* are based on *F*^2^, conventional *R*-factors *R* are based on *F*, with *F* set to zero for negative *F*^2^. The threshold expression of *F*^2^ > σ(*F*^2^) is used only for calculating *R*-factors(gt) *etc*. and is not relevant to the choice of reflections for refinement. *R*-factors based on *F*^2^ are statistically about twice as large as those based on *F*, and *R*- factors based on ALL data will be even larger.

### Fractional atomic coordinates and isotropic or equivalent isotropic displacement parameters (Å^2^)

**Table:**

	*x*	*y*	*z*	*U*_iso_*/*U*_eq_	
O1	0.65937 (15)	0.64947 (11)	0.44833 (6)	0.0133 (3)	
O2	0.13629 (15)	0.61728 (12)	0.40576 (6)	0.0146 (3)	
H2O	0.0696	0.5924	0.4355	0.018*	
O3	0.75905 (16)	0.31571 (12)	0.43788 (6)	0.0137 (3)	
O4	0.86693 (15)	0.54558 (12)	0.49981 (7)	0.0145 (3)	
O5	0.70889 (15)	0.63306 (12)	0.60424 (6)	0.0152 (3)	
H5O	0.6992	0.7126	0.6054	0.018*	
O6	0.58870 (16)	0.25696 (11)	0.54382 (6)	0.0128 (3)	
C1	0.2461 (2)	0.51713 (16)	0.38625 (8)	0.0117 (3)	
H1	0.1907	0.4359	0.3966	0.014*	
C2	0.2581 (2)	0.52931 (17)	0.31315 (8)	0.0161 (4)	
H2A	0.1461	0.5145	0.2943	0.019*	
H2B	0.2914	0.6158	0.3023	0.019*	
C3	0.3817 (2)	0.43951 (19)	0.28259 (8)	0.0175 (4)	
H3A	0.3854	0.4548	0.2356	0.021*	
H3B	0.3422	0.3529	0.2894	0.021*	
C4	0.5584 (2)	0.45268 (18)	0.31027 (8)	0.0154 (4)	
C5	0.5441 (2)	0.43633 (16)	0.38459 (8)	0.0109 (3)	
H5	0.5023	0.3493	0.3910	0.013*	
C6	0.7153 (2)	0.44034 (16)	0.41879 (8)	0.0118 (3)	
H6	0.8011	0.4716	0.3877	0.014*	
C7	0.7053 (2)	0.53030 (16)	0.47533 (8)	0.0111 (3)	
C8	0.5752 (2)	0.49180 (16)	0.52654 (8)	0.0108 (3)	
C9	0.4051 (2)	0.46841 (16)	0.49127 (8)	0.0096 (3)	
H9	0.3899	0.3760	0.4878	0.012*	
C10	0.4175 (2)	0.52120 (15)	0.42116 (8)	0.0104 (3)	
C11	0.2560 (2)	0.51986 (16)	0.53022 (8)	0.0129 (3)	
H11A	0.1528	0.4783	0.5150	0.015*	
H11B	0.2447	0.6101	0.5209	0.015*	
C12	0.2689 (2)	0.50260 (18)	0.60383 (8)	0.0147 (4)	
H12A	0.2307	0.4175	0.6152	0.018*	
H12B	0.1935	0.5629	0.6252	0.018*	
C13	0.4496 (2)	0.52170 (17)	0.62953 (8)	0.0138 (3)	
H13	0.4503	0.5653	0.6721	0.017*	
C14	0.5502 (2)	0.59337 (16)	0.57895 (8)	0.0124 (3)	
H14	0.4851	0.6658	0.5618	0.015*	
C15	0.6290 (2)	0.37800 (16)	0.56886 (8)	0.0121 (3)	
H15	0.7524	0.3825	0.5763	0.014*	
C16	0.5398 (2)	0.39814 (17)	0.63254 (8)	0.0134 (3)	
C17	0.5401 (2)	0.31766 (18)	0.68075 (9)	0.0172 (4)	
H17A	0.4786	0.3353	0.7188	0.021*	
H17B	0.6018	0.2422	0.6774	0.021*	
C18	0.6668 (2)	0.34633 (19)	0.28388 (9)	0.0206 (4)	
H18A	0.6680	0.3500	0.2368	0.025*	
H18B	0.6205	0.2661	0.2978	0.025*	
H18C	0.7811	0.3550	0.3002	0.025*	
C19	0.6374 (3)	0.57664 (19)	0.28789 (9)	0.0206 (4)	
H19A	0.5595	0.6453	0.2962	0.025*	
H19B	0.6613	0.5722	0.2417	0.025*	
H19C	0.7415	0.5911	0.3116	0.025*	
C20	0.4872 (2)	0.65416 (16)	0.42727 (9)	0.0124 (3)	
H20A	0.4802	0.6970	0.3851	0.015*	
H20B	0.4198	0.7020	0.4587	0.015*	
H3O	0.858 (4)	0.310 (2)	0.4432 (12)	0.028 (7)*	
H4O	0.860 (3)	0.577 (2)	0.5363 (12)	0.031 (7)*	
H6O	0.641 (3)	0.254 (2)	0.5097 (11)	0.015 (5)*	

### Atomic displacement parameters (Å^2^)

**Table:**

	*U*^11^	*U*^22^	*U*^33^	*U*^12^	*U*^13^	*U*^23^
O1	0.0098 (5)	0.0100 (6)	0.0200 (6)	−0.0011 (5)	−0.0014 (5)	0.0025 (5)
O2	0.0112 (6)	0.0128 (6)	0.0199 (6)	0.0031 (5)	0.0006 (5)	0.0017 (5)
O3	0.0103 (6)	0.0113 (6)	0.0195 (6)	0.0023 (5)	−0.0001 (5)	0.0013 (5)
O4	0.0082 (5)	0.0171 (6)	0.0180 (6)	−0.0010 (5)	−0.0009 (5)	−0.0017 (5)
O5	0.0142 (6)	0.0120 (6)	0.0195 (6)	−0.0022 (5)	−0.0040 (5)	−0.0024 (5)
O6	0.0134 (6)	0.0101 (6)	0.0147 (6)	−0.0004 (5)	0.0021 (5)	−0.0012 (5)
C1	0.0094 (7)	0.0102 (8)	0.0153 (8)	0.0010 (7)	−0.0013 (7)	0.0001 (6)
C2	0.0149 (8)	0.0188 (9)	0.0145 (8)	0.0016 (8)	−0.0034 (7)	0.0028 (7)
C3	0.0176 (9)	0.0227 (10)	0.0123 (8)	0.0021 (8)	−0.0012 (7)	0.0004 (7)
C4	0.0145 (8)	0.0192 (9)	0.0126 (8)	0.0018 (8)	0.0014 (7)	0.0005 (7)
C5	0.0107 (8)	0.0096 (8)	0.0125 (7)	0.0000 (7)	0.0002 (6)	−0.0001 (6)
C6	0.0097 (7)	0.0111 (8)	0.0146 (8)	0.0005 (6)	0.0011 (6)	0.0014 (7)
C7	0.0077 (7)	0.0105 (8)	0.0151 (8)	0.0001 (6)	−0.0010 (6)	0.0013 (7)
C8	0.0090 (7)	0.0104 (8)	0.0129 (8)	0.0005 (6)	−0.0014 (6)	0.0002 (6)
C9	0.0078 (7)	0.0092 (8)	0.0119 (7)	0.0002 (6)	−0.0003 (6)	0.0002 (6)
C10	0.0085 (7)	0.0096 (8)	0.0131 (8)	0.0005 (6)	0.0000 (6)	−0.0004 (6)
C11	0.0097 (8)	0.0133 (8)	0.0156 (8)	0.0011 (7)	0.0007 (7)	−0.0001 (7)
C12	0.0101 (8)	0.0190 (9)	0.0151 (8)	0.0014 (7)	0.0023 (7)	−0.0007 (7)
C13	0.0143 (8)	0.0139 (8)	0.0131 (8)	−0.0011 (7)	−0.0002 (7)	−0.0024 (7)
C14	0.0108 (7)	0.0115 (8)	0.0149 (8)	0.0000 (6)	−0.0007 (6)	−0.0013 (7)
C15	0.0104 (7)	0.0102 (8)	0.0156 (8)	−0.0008 (7)	−0.0017 (6)	−0.0003 (7)
C16	0.0105 (8)	0.0153 (9)	0.0143 (8)	−0.0022 (7)	−0.0017 (6)	−0.0020 (7)
C17	0.0173 (9)	0.0183 (9)	0.0159 (8)	0.0003 (8)	−0.0003 (7)	−0.0006 (7)
C18	0.0208 (9)	0.0258 (10)	0.0151 (8)	0.0043 (9)	0.0021 (8)	−0.0030 (8)
C19	0.0187 (9)	0.0256 (10)	0.0175 (8)	−0.0005 (8)	0.0041 (8)	0.0059 (8)
C20	0.0087 (7)	0.0110 (8)	0.0174 (8)	0.0008 (6)	−0.0012 (6)	0.0019 (7)

### Geometric parameters (Å, °)

**Table:**

O1—C7	1.442 (2)	C8—C14	1.553 (2)
O1—C20	1.447 (2)	C8—C15	1.564 (2)
O2—C1	1.445 (2)	C8—C9	1.566 (2)
O2—H2O	0.8580	C9—C11	1.543 (2)
O3—C6	1.437 (2)	C9—C10	1.566 (2)
O3—H3O	0.80 (3)	C9—H9	1.0000
O4—C7	1.399 (2)	C10—C20	1.535 (2)
O4—H4O	0.83 (3)	C11—C12	1.544 (2)
O5—C14	1.439 (2)	C11—H11A	0.9900
O5—H5O	0.8566	C11—H11B	0.9900
O6—C15	1.434 (2)	C12—C13	1.555 (2)
O6—H6O	0.83 (2)	C12—H12A	0.9900
C1—C2	1.527 (2)	C12—H12B	0.9900
C1—C10	1.552 (2)	C13—C16	1.509 (2)
C1—H1	1.0000	C13—C14	1.530 (2)
C2—C3	1.519 (3)	C13—H13	1.0000
C2—H2A	0.9900	C14—H14	1.0000
C2—H2B	0.9900	C15—C16	1.519 (2)
C3—C4	1.533 (3)	C15—H15	1.0000
C3—H3A	0.9900	C16—C17	1.322 (2)
C3—H3B	0.9900	C17—H17A	0.9500
C4—C18	1.533 (3)	C17—H17B	0.9500
C4—C19	1.543 (3)	C18—H18A	0.9800
C4—C5	1.558 (2)	C18—H18B	0.9800
C5—C6	1.543 (2)	C18—H18C	0.9800
C5—C10	1.559 (2)	C19—H19A	0.9800
C5—H5	1.0000	C19—H19B	0.9800
C6—C7	1.522 (2)	C19—H19C	0.9800
C6—H6	1.0000	C20—H20A	0.9900
C7—C8	1.545 (2)	C20—H20B	0.9900
			
C7—O1—C20	113.03 (13)	C20—C10—C1	112.68 (13)
C1—O2—H2O	110.4	C20—C10—C5	110.23 (13)
C6—O3—H3O	110.8 (18)	C1—C10—C5	109.26 (13)
C7—O4—H4O	108.3 (18)	C20—C10—C9	106.35 (14)
C14—O5—H5O	103.0	C1—C10—C9	111.64 (13)
C15—O6—H6O	103.4 (17)	C5—C10—C9	106.48 (13)
O2—C1—C2	104.68 (14)	C9—C11—C12	115.16 (14)
O2—C1—C10	112.67 (13)	C9—C11—H11A	108.5
C2—C1—C10	114.03 (14)	C12—C11—H11A	108.5
O2—C1—H1	108.4	C9—C11—H11B	108.5
C2—C1—H1	108.4	C12—C11—H11B	108.5
C10—C1—H1	108.4	H11A—C11—H11B	107.5
C3—C2—C1	113.72 (15)	C11—C12—C13	112.73 (14)
C3—C2—H2A	108.8	C11—C12—H12A	109.0
C1—C2—H2A	108.8	C13—C12—H12A	109.0
C3—C2—H2B	108.8	C11—C12—H12B	109.0
C1—C2—H2B	108.8	C13—C12—H12B	109.0
H2A—C2—H2B	107.7	H12A—C12—H12B	107.8
C2—C3—C4	112.67 (15)	C16—C13—C14	102.54 (14)
C2—C3—H3A	109.1	C16—C13—C12	110.09 (14)
C4—C3—H3A	109.1	C14—C13—C12	108.62 (14)
C2—C3—H3B	109.1	C16—C13—H13	111.7
C4—C3—H3B	109.1	C14—C13—H13	111.7
H3A—C3—H3B	107.8	C12—C13—H13	111.7
C3—C4—C18	108.61 (15)	O5—C14—C13	111.21 (14)
C3—C4—C19	110.12 (15)	O5—C14—C8	110.47 (13)
C18—C4—C19	107.50 (15)	C13—C14—C8	101.39 (13)
C3—C4—C5	107.07 (14)	O5—C14—H14	111.1
C18—C4—C5	108.20 (15)	C13—C14—H14	111.1
C19—C4—C5	115.19 (15)	C8—C14—H14	111.1
C6—C5—C4	112.82 (14)	O6—C15—C16	109.80 (14)
C6—C5—C10	109.63 (13)	O6—C15—C8	116.06 (13)
C4—C5—C10	117.72 (14)	C16—C15—C8	104.45 (14)
C6—C5—H5	105.2	O6—C15—H15	108.8
C4—C5—H5	105.2	C16—C15—H15	108.8
C10—C5—H5	105.2	C8—C15—H15	108.8
O3—C6—C7	112.87 (14)	C17—C16—C13	127.20 (17)
O3—C6—C5	108.51 (13)	C17—C16—C15	124.52 (17)
C7—C6—C5	109.07 (13)	C13—C16—C15	108.26 (14)
O3—C6—H6	108.8	C16—C17—H17A	120.0
C7—C6—H6	108.8	C16—C17—H17B	120.0
C5—C6—H6	108.8	H17A—C17—H17B	120.0
O4—C7—O1	105.86 (13)	C4—C18—H18A	109.5
O4—C7—C6	107.85 (13)	C4—C18—H18B	109.5
O1—C7—C6	105.90 (13)	H18A—C18—H18B	109.5
O4—C7—C8	113.82 (14)	C4—C18—H18C	109.5
O1—C7—C8	109.42 (13)	H18A—C18—H18C	109.5
C6—C7—C8	113.43 (14)	H18B—C18—H18C	109.5
C7—C8—C14	112.50 (14)	C4—C19—H19A	109.5
C7—C8—C15	114.19 (13)	C4—C19—H19B	109.5
C14—C8—C15	100.83 (13)	H19A—C19—H19B	109.5
C7—C8—C9	107.83 (13)	C4—C19—H19C	109.5
C14—C8—C9	109.18 (13)	H19A—C19—H19C	109.5
C15—C8—C9	112.19 (13)	H19B—C19—H19C	109.5
C11—C9—C10	114.06 (13)	O1—C20—C10	109.79 (13)
C11—C9—C8	111.66 (13)	O1—C20—H20A	109.7
C10—C9—C8	108.81 (13)	C10—C20—H20A	109.7
C11—C9—H9	107.3	O1—C20—H20B	109.7
C10—C9—H9	107.3	C10—C20—H20B	109.7
C8—C9—H9	107.3	H20A—C20—H20B	108.2
			
O2—C1—C2—C3	174.61 (14)	C6—C5—C10—C20	55.37 (17)
C10—C1—C2—C3	51.0 (2)	C4—C5—C10—C20	−75.36 (18)
C1—C2—C3—C4	−56.8 (2)	C6—C5—C10—C1	179.71 (13)
C2—C3—C4—C18	171.78 (15)	C4—C5—C10—C1	48.98 (19)
C2—C3—C4—C19	−70.76 (19)	C6—C5—C10—C9	−59.58 (16)
C2—C3—C4—C5	55.2 (2)	C4—C5—C10—C9	169.69 (14)
C3—C4—C5—C6	176.91 (14)	C11—C9—C10—C20	76.19 (16)
C18—C4—C5—C6	60.02 (19)	C8—C9—C10—C20	−49.19 (16)
C19—C4—C5—C6	−60.3 (2)	C11—C9—C10—C1	−47.07 (18)
C3—C4—C5—C10	−53.8 (2)	C8—C9—C10—C1	−172.46 (13)
C18—C4—C5—C10	−170.73 (14)	C11—C9—C10—C5	−166.25 (14)
C19—C4—C5—C10	69.0 (2)	C8—C9—C10—C5	68.36 (15)
C4—C5—C6—O3	−105.74 (16)	C10—C9—C11—C12	−162.27 (14)
C10—C5—C6—O3	120.97 (14)	C8—C9—C11—C12	−38.4 (2)
C4—C5—C6—C7	130.94 (15)	C9—C11—C12—C13	38.2 (2)
C10—C5—C6—C7	−2.36 (18)	C11—C12—C13—C16	−93.00 (18)
C20—O1—C7—O4	−173.34 (13)	C11—C12—C13—C14	18.5 (2)
C20—O1—C7—C6	72.31 (16)	C16—C13—C14—O5	−73.63 (16)
C20—O1—C7—C8	−50.29 (17)	C12—C13—C14—O5	169.86 (13)
O3—C6—C7—O4	67.47 (17)	C16—C13—C14—C8	43.82 (16)
C5—C6—C7—O4	−171.83 (13)	C12—C13—C14—C8	−72.69 (16)
O3—C6—C7—O1	−179.55 (13)	C7—C8—C14—O5	−50.02 (18)
C5—C6—C7—O1	−58.86 (16)	C15—C8—C14—O5	72.05 (16)
O3—C6—C7—C8	−59.54 (18)	C9—C8—C14—O5	−169.69 (13)
C5—C6—C7—C8	61.16 (18)	C7—C8—C14—C13	−168.00 (14)
O4—C7—C8—C14	63.85 (19)	C15—C8—C14—C13	−45.94 (16)
O1—C7—C8—C14	−54.34 (17)	C9—C8—C14—C13	72.33 (16)
C6—C7—C8—C14	−172.34 (14)	C7—C8—C15—O6	−87.44 (17)
O4—C7—C8—C15	−50.29 (19)	C14—C8—C15—O6	151.70 (14)
O1—C7—C8—C15	−168.48 (13)	C9—C8—C15—O6	35.65 (19)
C6—C7—C8—C15	73.52 (18)	C7—C8—C15—C16	151.54 (14)
O4—C7—C8—C9	−175.70 (13)	C14—C8—C15—C16	30.67 (16)
O1—C7—C8—C9	66.11 (16)	C9—C8—C15—C16	−85.38 (16)
C6—C7—C8—C9	−51.89 (18)	C14—C13—C16—C17	156.98 (18)
C7—C8—C9—C11	−139.59 (14)	C12—C13—C16—C17	−87.6 (2)
C14—C8—C9—C11	−17.08 (18)	C14—C13—C16—C15	−24.71 (17)
C15—C8—C9—C11	93.82 (16)	C12—C13—C16—C15	90.74 (16)
C7—C8—C9—C10	−12.81 (17)	O6—C15—C16—C17	49.2 (2)
C14—C8—C9—C10	109.70 (15)	C8—C15—C16—C17	174.27 (17)
C15—C8—C9—C10	−139.41 (14)	O6—C15—C16—C13	−129.19 (14)
O2—C1—C10—C20	−41.24 (19)	C8—C15—C16—C13	−4.09 (17)
C2—C1—C10—C20	77.89 (18)	C7—O1—C20—C10	−16.70 (18)
O2—C1—C10—C5	−164.13 (13)	C1—C10—C20—O1	−168.75 (13)
C2—C1—C10—C5	−45.00 (19)	C5—C10—C20—O1	−46.40 (18)
O2—C1—C10—C9	78.35 (17)	C9—C10—C20—O1	68.64 (16)
C2—C1—C10—C9	−162.51 (14)		

### Hydrogen-bond geometry (Å, °)

**Table:**

*D*—H···*A*	*D*—H	H···*A*	*D*···*A*	*D*—H···*A*
O3—H3O···O6^i^	0.80 (3)	2.00 (3)	2.7761 (18)	164 (3)
O4—H4O···O5	0.83 (3)	1.95 (3)	2.6806 (18)	146 (2)
O5—H5O···O2^ii^	0.86	1.90	2.7455 (18)	167
O6—H6O···O3	0.83 (2)	1.88 (2)	2.6642 (18)	157 (2)

Symmetry codes: (i) *x*+1/2, −*y*+1/2, −*z*+1; (ii) *x*+1/2, −*y*+3/2, −*z*+1.
